# Maturational trajectory of fusiform gyrus neural activity when viewing faces: From 4 months to 4 years old

**DOI:** 10.3389/fnhum.2022.917851

**Published:** 2022-08-12

**Authors:** Yuhan Chen, Olivia Allison, Heather L. Green, Emily S. Kuschner, Song Liu, Mina Kim, Michelle Slinger, Kylie Mol, Taylor Chiang, Luke Bloy, Timothy P. L. Roberts, J. Christopher Edgar

**Affiliations:** ^1^Lurie Family Foundations MEG Imaging Center, Children’s Hospital of Philadelphia, Philadelphia, PA, United States; ^2^Department of Psychiatry, Perelman School of Medicine, University of Pennsylvania, Philadelphia, PA, United States; ^3^Department of Radiology, Perelman School of Medicine, University of Pennsylvania, Philadelphia, PA, United States

**Keywords:** MEG (magnetoencephalography), infants, preschoolers, maturation, fusiform gyrus, face, social

## Abstract

Infant and young child electrophysiology studies have provided information regarding the maturation of face-encoding neural processes. A limitation of previous research is that very few studies have examined face-encoding processes in children 12–48 months of age, a developmental period characterized by rapid changes in the ability to encode facial information. The present study sought to fill this gap in the literature *via* a longitudinal study examining the maturation of a primary node in the face-encoding network—the left and right fusiform gyrus (FFG). Whole-brain magnetoencephalography (MEG) data were obtained from 25 infants with typical development at 4–12 months, and with follow-up MEG exams every ∼12 months until 3–4 years old. Children were presented with color images of Face stimuli and visual noise images (matched on spatial frequency, color distribution, and outer contour) that served as Non-Face stimuli. Using distributed source modeling, left and right face-sensitive FFG evoked waveforms were obtained from each child at each visit, with face-sensitive activity identified *via* examining the difference between the Non-Face and Face FFG timecourses. Before 24 months of age (Visits 1 and 2) the face-sensitive FFG M290 response was the dominant response, observed in the left and right FFG ∼250–450 ms post-stimulus. By 3–4 years old (Visit 4), the left and right face-sensitive FFG response occurred at a latency consistent with a face-sensitive M170 response ∼100–250 ms post-stimulus. Face-sensitive left and right FFG peak latencies decreased as a function of age (with age explaining greater than 70% of the variance in face-sensitive FFG latency), and with an adult-like FFG latency observed at 3–4 years old. Study findings thus showed face-sensitive FFG maturational changes across the first 4 years of life. Whereas a face-sensitive M290 response was observed under 2 years of age, by 3–4 years old, an adult-like face-sensitive M170 response was observed bilaterally. Future studies evaluating the maturation of face-sensitive FFG activity in infants at risk for neurodevelopmental disorders are of interest, with the present findings suggesting age-specific face-sensitive neural markers of *a priori* interest.

## Introduction

Humans have domain-specific strategies for encoding facial information, an ability that has evolved across millennium (Kanwisher, 2000; McKone et al., 2007; Baek et al., 2021). There is a sensitive period for encoding facial information, with the ability to recognize faces as salient visual stimuli developing during infancy (de Heering and Rossion, 2015; Di Lorenzo et al., 2020) and with this ability continually refined throughout early childhood (Taylor et al., 2004). Accurately identifying facial social cues is considered essential to acquiring social communication skills throughout early childhood and adolescence (Dawson et al., 2004; Sperdin et al., 2018).

Behavioral studies have demonstrated that face-encoding processes undergo marked maturational change throughout infancy and toddlerhood. For example, although newborns do not show a spontaneous visual preference for human vs. primate faces, newborns do discriminate human and primate faces from inverted faces or non-face stimuli (Farroni et al., 2005; Johnson et al., 2005; Di Giorgio et al., 2012; Grossman and Johnson, 2013). Between 6 and 9 months of age infants develop a preference for human faces (Pascalis et al., 2002, 2011; Johnson et al., 2015), and by 10 months of age infants holistically process facial information, evidenced by their ability to identify an incorrect placement of the eyes and mouth (Schwarzer et al., 2007). Studies have suggested that across time children apply different strategies for interpreting featural or configural facial information (Carey, 1992), with children processing more information from facial features as well as processing facial information more efficiently as they age (Flin, 1985; Baenninger, 1994; Chung and Thomson, 1995).

Although pediatric behavioral studies provide insight regarding the development of face-processing abilities, studying face processing from birth to preschool is challenging due to the limited and variable ability of infants, toddlers and preschoolers to perform behavioral face recognition tasks. Electroencephalography (EEG) and magnetoencephalography (MEG) are promising non-invasive methods for studying how the brain encodes facial information in real time. As reviewed below, the maturation of key neural correlates involved in face encoding from infancy through preschool is just beginning to be understood. The present longitudinal MEG study sought to address gaps in our understanding by measuring the neural activity in left and right fusiform gyrus (FFG; brain regions key to processing faces—see following paragraphs) associated with face encoding.

### Pediatric electrophysiology face-processing studies

In adults, an evoked response sensitive to face stimuli occurs ∼170 ms following stimulus onset, with this response referred to as the N170 in EEG (Bentin et al., 1996; Rebai et al., 2001; de Haan et al., 2002; Pizzagalli et al., 2002; Nakashima et al., 2008; Caharel and Rossion, 2021) and the M170 in MEG (Halgren et al., 2000; Liu et al., 2000; Watanabe et al., 2005; Xu et al., 2005; Gao et al., 2013; Monroe et al., 2013). The adult N170/M170 is termed “face-sensitive” as it is of larger amplitude and shorter latency in response to faces compared to objects, inverted faces, or scrambled faces (Bentin et al., 1996; Eimer, 2000a,b,c; Rossion et al., 2000; de Haan et al., 2002; Itier and Taylor, 2004). EEG studies find that the N170 is largest over occipital and temporal scalp sensors. MEG studies report that M170 activity localizes to the left and right FFG (Halgren et al., 2000; Deffke et al., 2007; Henson et al., 2009; Monroe et al., 2013).

In infant and young child EEG studies, the evoked responses associated with processing faces are referred to as the N290 (de Haan et al., 2003; Peykarjou and Hoehl, 2013; Peykarjou et al., 2013; Guy et al., 2016; Conte et al., 2020; Di Lorenzo et al., 2020) and the P400 (de Haan and Nelson, 1999; de Haan et al., 2003; Halit et al., 2003; Peykarjou and Hoehl, 2013; Peykarjou et al., 2013; Hoehl, 2015; Munsters et al., 2019). The N290 is a negative peak at ∼250–350 ms, and the P400 is a positive peak at ∼300–600 ms (Peykarjou et al., 2013). N290 amplitude differences between faces and meaningless patterns or objects are frequently reported (de Haan and Nelson, 1999; Halit et al., 2004; Gliga and Dehaene-Lambertz, 2007; Kouider et al., 2013; Peykarjou and Hoehl, 2013). The latency of the N290 (and its magnetic counterpart the M290) decreases from 3 to 12 months of age (de Haan et al., 2003; Halit et al., 2003). The N290 is hypothesized to be a precursor of the N170 (de Haan et al., 2003), with research showing that the N290 amplitude in a 12-month-old is modulated by upright and inverted faces the same way as the adult N170 (Halit et al., 2003). The P400 has not been studied as frequently as the N290, possibly because the amplitude of P400 has not been consistently observed to be sensitive to face stimuli (see review in Conte et al., 2020), making the role of P400 in face processing is somewhat ambiguous.

Some studies have examined both N290 and P400 responses in younger infants. Two studies have found that whereas an N290 amplitude difference between upright vs. inverted face stimulus is not observed in 3–5 month old infants, P400 amplitude differences between upright vs. inverted faces are observed in this age range (de Haan et al., 2002; Halit et al., 2003). Comparing brain responses to faces vs. toys in 4.5–7.5-month-old infants, Guy et al. (2016) found larger N290 responses to faces than toys, and a larger P400 amplitude to toys than faces. In a longitudinal infant study of 5–10 month old infants, Di Lorenzo et al. (2020) examined the development of three face-sensitive EEG components: P1, N290, and P400. Compared to the P400 and P1, N290 was the dominant response, with a face-sensitive N290 response observed in almost all infants at 5 and 10 months of age. Across the majority of infants, the N290 amplitude was larger to face vs. house stimuli at Visit 1 (5 months) and Visit 2 (10 months). To date, study findings suggest that the N290 is the primary face-sensitive response in infants. Although it is suggested that the N170 emerges from the integration of the N290 and P400 (Conte et al., 2020), there is currently no evidence showing that the P400 (latency or amplitude) changes as infants age or demonstrating the development of N290 and P400 into N170.

Research suggests that the N290 and the P400 have distinct cortical generators. Using volumetric current density reconstruction and sLORETA (Pascual-Marqui et al., 1994; Pascual-Marqui, 2002), Guy and colleagues (Guy et al., 2016) showed that whereas the N290 localizes to FFG and temporal pole, the P400 generators are broadly distributed across midline frontal, parietal, temporal and occipital regions. In a study from our group, using a dedicated infant MEG system to examine left and right FFG neural activity in infants 3–24 months (cross-sectional study) (Chen et al., 2021), right FFG peak latency (FFG M290 responses) decreased as a function of age, with stronger FFG activity observed in response to face than non-face stimuli ∼250–400 ms post-stimulus.

EEG and MEG pediatric face-encoding studies have focused on infants under 12 months or school-age children 5 years and older, with little known about face neural processes in children 12–48 months of age. Bhavnani et al. (2021) reviewed EEG measures of cognitive and social development in 2–5-year-old children and identified seven papers reporting on face processing. In two studies most of the children were under 4 years old (Carver et al., 2003; Peykarjou et al., 2013), one study included children 4–5 years old (Taylor et al., 2001), and the remaining four studies included children 5 + years old. Across studies, in children 4 years and older, the EEG N170 and P1 were most often reported to be sensitive to face stimuli. As an example, Taylor et al. (1999, 2001) observed a face-sensitive N170 in children 4–15 years old, but with a smaller amplitude and later latency than the adult N170. Above EEG findings indicate that face-encoding abilities evolve throughout childhood, with EEG findings showing adult-like processing of the eyes by ∼11 years old, and with whole-face encoding processes continuing to mature until adulthood (Taylor et al., 2001).

Consistent with the EEG review from Bhavnani and colleagues, MEG studies have reported adult-like M170 responses by 4 years of age. Examining one 4-year-old child (He et al., 2014) as well as a cross-sectional group of children 3–6 years old (*N* = 15) (He et al., 2015), an “M170” response showed a difference in strength between faces and scrambled faces. They noted, however, that the response occurred at a latency later than the adult M170 response. This is likely due to the fact that their cohort included children as young as 3 years old. Their findings, as well as previous EEG face-encoding studies, draw attention to the need for longitudinal studies to better understand the development of FFG face-encoding neural activity from birth to 4 years old.

### Study aims and hypotheses

The present longitudinal study examined changes to the FFG M290 and M170 response from 4 months to 4 years old. Results from previous cross-sectional studies suggest that the N290 and the N170 are modulated by the same stimulus contrast (e.g., face vs. objects), and that the N290 eventually “becomes” the N170 (Taylor et al., 1999, 2001; de Haan et al., 2003; Halit et al., 2003; He et al., 2014, 2015). As the EEG N290 response is the most consistently observed response to faces in infants (Di Lorenzo et al., 2020), and given that fMRI and MEG studies demonstrate strong FFG activity when encoding faces (Kanwisher et al., 1997; Haxby et al., 1999; Watanabe et al., 1999; Halgren et al., 2000; Grill-Spector et al., 2004; Pourtois et al., 2010), the present study focused on FFG neural activity. MEG data during a face-encoding exam were obtained from infants 4–12 months old, with follow-up data collected every ∼12 months until 3–4 years, for a total of 4 visits. It was hypothesized that the latency of the left and right FFG face-sensitive response (i.e., showing a difference between face and non-face stimuli) would decrease as a function of age, with M290 responses observed at Visits 1 and 2, and with an “adult-like” FFG M170 response observed at Visits 3 and 4. Study findings would thus provide FFG face-processing growth curves.

## Materials and methods

### Participants

Longitudinal MEG data were acquired from 22 typically developing infants (13 males, Visit 1: 4–12 months), with follow-up MEG measures obtained approximately every 12 months (Visit 2: 18–24 months, Visit 3: 30–36 months, and Visit 4: 41–53 months). Evaluable Visit 1 data were obtained from 21 children, at Visit 2 from 18 children, at Visit 3 from 14 children, and at Visit 4 from 13 children (Table 1). As shown in Table 2, MEG data at all 4 visits were obtained from 11 children, at 3 visits from 2 children, at 2 visits from 7 children, and at 1 visit from 2 children. Due to the COVID-19 pandemic, some Visit 4 scans were canceled or delayed, and thus some Visit 4 data were collected after 48 months of age. Of note, Chen et al. (2021) reported single-time-point data from a subset of children included in the present study.

**TABLE 1:** Demographic information and developmental milestone scores at each visit.

	Age (months) mean (*SD*)	Sex (male/female)	MSEL ELC mean (*SD*)	VABS social mean (*SD*)	VABS ABC mean (*SD*)
Visit 1 (*N* = 21)	8.38 (2.10)	13 M/8 F	103.50 (25.76)	99.84 (6.67)	99.42 (3.95)
Visit 2 (*N* = 18)	20.71 (2.12)	12 M/6 F	99.21 (15.86)	91.56 (5.50)	92.56 (6.75)
Visit 3 (*N* = 14)	33.34 (2.20)	9 M/5 F	107.41 (13.17)	93.75 (8.93)	94.00 (9.90)
Visit 4 (*N* = 13)	46.50 (3.51)	9 M/4 F	106.44 (15.28)	98.42 (9.20)	98.08 (10.04)

**TABLE 2:** Face vs. Non-Face FFG peak latency for each child at each visit.

	Age (months)				L-FFG M290 Latency (ms)		R-FFG M290 Latency (ms)		L-FFG M170 Latency (ms)		R-FFG M170 Latency (ms)	
Subject	Visit 1 (*N* = 21)	Visit 2 (*N* = 18)	Visit 3 (*N* = 14)	Visit 4 (*N* = 13)	Visit 1 (81%)	Visit 2 (72%)	Visit 1 (100%)	Visit 2 (83%)	Visit 3 (64%)	Visit 4 (86%)	Visit 3 (85%)	Visit 4 (92%)
S1	4.0	23.8	Missed	Missed	absent	203	408	212	Missed	Missed	Missed	Missed
S2	8.0	19.8	Missed	Missed	311	absent	334	268	Missed	Missed	Missed	Missed
S3	6.1	Missed	Missed	Missed	360	Missed	382	Missed	Missed	Missed	Missed	Missed
S4	10.7	22.0	36.2	48.9	426	221	349	Absent	Absent	Absent	271	134
S5	10.7	23.2	Missed	Missed	Absent	324	289	Absent	Missed	Missed	Missed	Missed
S6	11.1	21.5	34.6	44.4	Absent	377	411	284	269	Absent	Absent	228
S7	Missed	Missed	35.4	53.9	Missed	Missed	Missed	Missed	161	206	133	136
S8	7.0	18.4	31.0	48.5	351	349	378	276	Absent	208	183	171
S9	12.0	24.7	37.4	Missed	346	absent	240	302	Absent	Missed	278	Missed
S10	9.0	Missed	Missed	Missed	415	Missed	339	Missed	Missed	Missed	Missed	Missed
S11	7.3	19.0	30.2	41.8	Absent	Absent	366	321	Absent	134	Absent	Absent
S12	12.0	22.3	34.8	52.1	302	336	379	260	227	140	159	132
S13	8.9	Missed	34.4	47.6	323	Missed	430	Missed	272	207	183	177
S14	6.3	16.7	Missed	Missed	351	297	313	330	Missed	Missed	Missed	Missed
S15	7.2	22.9	34.5	48.5	306	Absent	368	217	Absent	136	210	193
S16	8.9	18.9	Missed	Missed	365	341	294	291	Missed	Missed	Missed	Missed
S17	10.4	20.7	33.5	48.4	270	Absent	328	284	231	124	256	194
S18	7.6	20.6	32.3	43.4	341	314	404	265	148	167	162	142
S19	6.2	23.6	36.0	47.6	416	293	370	257	165	170	188	251
S20	7.4	20.5	30.4	43.2	362	319	377	Absent	Absent	134	227	217
S21	8.2	20.3	32.5	44.5	299	335	391	313	210	126	196	143
S22	9.3	19.2	Missed	Missed	357	393	374	264	Missed	Missed	Missed	Missed

“Absent” indicates that a stronger Face than Non-Face response was not observed; “Missed” indicates a missed visit, or unevaluable data. Under each Visit column, % indicates percentage of children with an M290 or M170 response.

Selection criteria were: (1) no seizure disorder in the child or an immediate family member; (2) no premature birth (later than 37 weeks gestation); (3) no non-removable metal in the body; (4) no known hearing or visual impairment; and (5) no concerns regarding language or developmental delay. Children were included or excluded based on parental report and review of medical records. The study was approved by the Children’s Hospital of Philadelphia IRB and all families gave written consent.

For each child at each visit, developmental assessments confirmed eligibility for typical development. Cognitive ability was assessed using the Mullen Scales of Early Learning (MSEL; Mullen, 1995). The MSEL provides a clinical assessment of verbal and non-verbal abilities for children from birth to 68 months. The MSEL includes Visual Reception, Fine Motor, Receptive Language, and Expressive Language domains. In addition, the Vineland Adaptive Behavior Scales-Third Edition (VABS-III; Sparrow et al., 2016) evaluated social and adaptive behavior, with socialization and adaptive behavior composite (ABC) standard scores obtained at each visit. Social ability was operationalized as the standard score of the Socialization Domain from the VABS, which includes the “Interpersonal Relationships,” and “Play and Leisure Time” subdomains. Table 1 shows demographic information as well as MSEL Early Learning Composite (ELC) scores and VABS social and ABC standardized scores. All children demonstrated typical cognitive, social, and adaptive behavior development, scoring within 2 standard deviations (SD) of the mean for each domain at each visit.

### Stimuli

**FIGURE 1:**
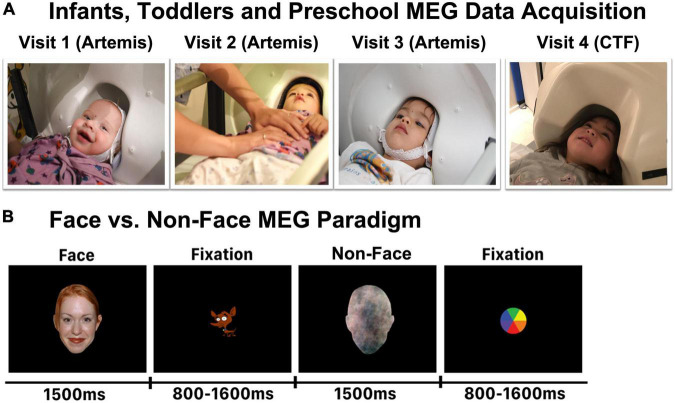
**(A)** Representative infants and young children in the infant MEG (Artemis) or the conventional MEG (CTF) helmet. Each child was scanned in the Artemis MEG system at Visits 1, 2, and 3, and in the CTF MEG system at Visit 4. **(B)** Face and Non-Face paradigm.

Stimuli consisted of 80 color images of Face stimuli (NimStim) and 80 matched visual noise images that served as Non-Face stimuli (Figure 1B). Face stimuli exhibiting a happy expression were selected from the NimStim Face Stimulus Set (Tottenham et al., 2009). Using the approach described in Halit et al. (2004), Non-Face stimuli were created to match the frequency content (spatial frequency), color distribution, and outer contour of the Face stimuli. Such Non-Face control stimuli are necessary, as studies have shown that face-selective responses in infants and toddlers can be due to stimuli differences in spatial frequency (Cassia et al., 2004; Simion et al., 2007; de Heering et al., 2008; Macchi Cassia et al., 2011; Simion and Giorgio, 2015).

It is of acknowledged that the control stimuli used in infant and young children face research remains a topic of debate. Although some studies have used houses or toys as control conditions, these control stimuli are generally not matched to the face stimuli with respect to low-level psychophysical properties such as spatial frequency. As described in Halit et al. (2004), this is a concern in infant studies, where such factors are known to influence the preference and processing of visual stimuli (Banks and Salapatek, 1981), as well as possibly engaging other neural regions or circuits involved in action planning (e.g., a 12-month-old infant who views an image of a toy they hope to grab) or showing stimuli not yet known to a child (e.g., a 3-month-old infant who views images of houses when they have not yet seen a house from the outside) (Kaufman et al., 2003).

With respect to the present study, it is also of note that images of non-face objects such as houses or toys have a vastly different importance to children 4 months old vs. 4 years old (and even across different 3–4-year-old children). As such, for the purposes of the present study, using toy or house objects as a contrast condition was not ideal given the goal of evaluating the maturation of FFG face-sensitive cortical responses from 4 months to 4 years. In the present study, Non-Face stimuli were produced by randomizing the phase spectrum of each face picture (overlaid on a head shape). Non-Face stimuli thus retained the amplitude and color spectra as well as the contour of the face stimulus, but were not identifiable as a face (see Figure 1B). All stimuli were presented against a black background with a horizontal visual angle of 12.6 degrees, a vertical angle of 18.9 degrees, and a viewing distance of 45 cm. Stimulus duration was 1,500 ms and the inter-stimulus interval varied randomly between 800 and 1,200 ms (Figure 1B). Face and Non-Face stimuli were randomly presented and no stimulus was repeated. Thus, each participant was shown 80 unique Face and 80 unique Non-Face stimuli.

### Magnetoencephalography data acquisition

At Visits 1, 2, and 3 (under 36 months of age; see Figure 1A), infant whole-head MEG data were recorded in a magnetically shielded room (MSR; Vacuumschmelze GmbH and Co., KG, Hanau, Germany) using Artemis 123 (Tristan Technologies Inc., San Diego, CA, United States) with a sampling rate of 5,000 and a 0.1 Hz high-pass filter. The Artemis 123 was designed for use with children from birth to 3 years of age (Roberts et al., 2014; Edgar et al., 2015). The Artemis system has 123 first-order axial gradiometers and a helmet circumference of 50 cm, which corresponds to the median head circumference of 36-month-old children in the U.S. The Artemis 123 employs a coil-in-vacuum sensor configuration to minimize the distance between the helmet surface and sensors (6–9 mm). During the MEG recording, the child’s head position was continuously monitored using 4 head position indicator (HPI) coils attached to a fabric cap that each child wore during the scan.

By Visit 4 (41–53 months; see Figure 1A), most children no longer fit in the infant MEG helmet. Thus, at Visit 4, whole-head MEG data were acquired in the same MSR using the 275 axial gradiometer CTF system (VSM MedTech, Coquitlam, BC) and with synthetic third-order gradiometer noise correction. CTF MEG data were acquired with a sampling rate of 1,200 Hz. The child’s head position was monitored using 3 HPIs attached to the scalp with continuous head localization applied.

To help keep the child calm and engaged during the MEG exam, a research assistant with experience scanning infants and young children stood next to the child and helped the parent keep the child calm and alert during the exam. With parental permission, children younger than 9 months were swaddled to reduce motion. Several strategies were used to make sure MEG data were obtained while the infant was looking at the stimuli and thus to optimize the number of evaluable trials. First, when needed, the research assistant instructed the parent to provide a pacifier or bottle to reduce motion and to help keep the infant calm while watching the video screen. Second, the paradigm was designed to keep the infant’s attention throughout the exam, with “attention grabbers” (e.g., animated animals with sound) presented in-between the Face or Non-Face stimuli. Third, when an infant was fussy and unable to attend to the stimuli, or when there was excessive motion, the task was paused, with toys or bottle/pacifier provided during the break (and with the infant’s head remaining in the MEG helmet). Presentation of the stimuli was resumed only once the infant was able to again pay attention to the stimuli. Fourth, the task was paused if the infant began to fall asleep. Use of the above procedures minimized the number of excluded trials. In addition to the above, the time when the child was not attending to the stimuli during the recording was noted by the study staff (e.g., starting to fall asleep) and this data manually removed.

Of note, the Artemis and CTF MEG systems were in the same MSR and used the same hardware for stimulus presentation (projector, presentation computer). The size of the stimulus screens used for CTF and Artemis are the same, and the children were scanned in the supine position at all visits, viewing the same sized Face and Non-Face stimuli and with the viewing distance and visual angle the same in both MEG systems.

For each child at each time point, MEG data were co-registered to an age-appropriate infant or young child MRI template (O’Reilly et al., 2021). An affine transformation accommodated global scale differences between the child’s anatomy and the atlas. Before MEG data acquisition, each child’s head shape, anatomical landmarks (nasion, right, and left preauriculars), as well as the location of the HPI coils were digitized using the FastSCAN System (Polhemus, Colchester, VT). The points representing the shape of the child’s head (>500 points) were used to co-register the MEG and the MRI template surface (warped to fit the MRI surface).

### Magnetic source analyses

Artemis and CTF MEG data were analyzed using Brainstorm (Tadel et al., 2011).^1^ All MEG data were down-sampled to 1,000 Hz and then band-pass filtered from 3 to 55 Hz (low transition: 1.5–3.0 Hz, high transition: 55–63.25 Hz, stopband attenuation: 60 Hz), and with a 60 Hz notch filter applied. Heartbeat artifact was removed *via* independent component analyses (ICA). Other artifacts (e.g., movement, muscle artifact) were visually identified and manually removed. During the scan, times when the participant was not attending to the stimuli were noted (e.g., crying, falling asleep) and this data were manually removed. In addition to removing data containing excessive artifacts due to motion or magnetic noise, trials with amplitudes exceeding 500 fT were excluded. The average number of artifact-free trials for the Face and Non-Face conditions at each time point are provided in Table 3. No subject had fewer than 30 trials for condition at any time point.

**TABLE 3:** Mean, range, and standard deviation of number of trials per condition at each time point.

	Face	Non-Face
	Mean (Range)/	Mean (Range)/
	Standard Deviation	Standard Deviation
Visit 1 (*N* = 21)	65.81 (49–79)/7/46	66.10 (43–78)/9.23
Visit 2 (*N* = 18)	68.56 (54–80)/8.43	69.61 (54–79)/8.04
Visit 3 (*N* = 14)	66.10 (30–79)/16.37	66.79 (32–80) 17.52
Visit 4 (*N* = 13)	74.77 (65–80)/4.75	73.77 (55–79)/7.25

**FIGURE 2:**
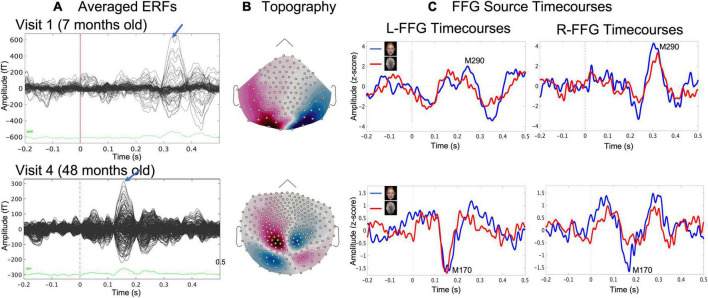
Example data from a representative child at Visit 1 (age 7 months, upper row) and at Visit 4 (age 48 months, lower row) showing **(A)** sensor event-related field (ERF) butterfly plots for the Face condition and **(B)** magnetic field sensor topography for the Face condition at the time where the blue arrow in **(A)** indicated the face-response peak latency (MEG sensors), and **(C)** evoked source timecourses for Face (blue) and Non-Face (red) conditions at left and right FFG.

Face and Non-Face event-related fields (ERFs; Figure 2A) were created by averaging epochs 200 ms pre-stimulus to 500 ms post-stimulus. Distributed source modeling provided estimates of neural activity throughout the brain. An advantage of using MEG to study early brain development is that MEG is much less sensitive than EEG to distortion of the volume current caused by open fontanels and sutures and to inaccurate estimates of skull conductivity (Lew et al., 2013; Chen et al., 2019). To calculate the MEG forward solution, an overlapping spheres head model was created for each participant. Whole-brain distributed modeling source reconstruction for Face and Non-Face ERFs were computed using Dynamical Statistical Parametric Mapping (dSPM; Dale et al., 2000) with constrained orientation estimating FFG activity associated with Face and Non-Face stimuli. For computing each child’s dSPM solution, an MEG noise covariance matrix for each child was obtained from an empty room recording immediately prior to the child’s scan. dSPM solutions were computed with normalization as part of the inverse routine based on the noise covariance, resulting in a Z-score map.

### Left and right fusiform gyrus source timecourses

For each child and at each visit, Face and Non-Face dSPM Z-score maps were created, and a left and right FFG (L-FFG and R-FFG) region-of-interest (ROI) was obtained based on the Desikan-Killiany Atlas (Desikan et al., 2006). For each child at each visit, L-FFG and R-FFG source timecourses were obtained by averaging the source strength from each vertex within the L-FFG and R-FFG ROI, separately for the Face and Non-Face conditions (Figure 2C). Left and right Non-Face FFG timecourses were subtracted from Face FFG timecourses, with the difference waveform used to identify FFG face-specific responses. For each child at each visit, L-FFG and R-FFG peak latency was identified as the time point showing the largest amplitude (magnetic field topography shown in Figure 2B) after the first positive peak (i.e., the M100, the magnetic counterpart of P1 response).

### Statistical analyses

Paired-sample *t*-tests examined Face vs. Non-Face left and right FFG amplitude timecourse differences at each sample from 0–500 ms post-stimulus. A cluster threshold of *p* < 0.05 for 20 ms+ family-wise correction was applied. Given changes to the FFG source timecourse morphology across time (changing responses and latencies), the Face vs. Non-Face FFG source timecourses were examined at each visit rather than statistically comparing timecourses across visits. A primary focus of the study was to determine the presence/absence of a L-FFG and R-FFG M290 and/or M170 response as well as the latency of the L-FFG and R-FFG M290 and M170 response at each visit.

Given missing data (e.g., one child with only Visit 1 and 3 data), mixed-effect models evaluated Face vs. Non-Face FFG peak latency changes as a function of age, with FFG peak latency the dependent variable, and with Visit (1, 2, 3, 4), Hemisphere (left, right), and their interactions entered as fixed effects variables, and subject entered as a random effect variable.

## Results

### Maturation of the fusiform gyrus face response

**FIGURE 3:**
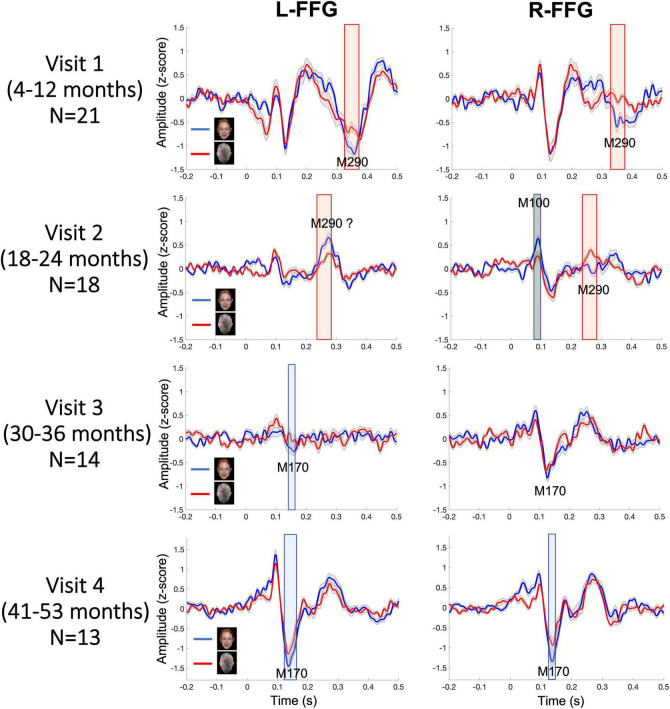
Grand average Face (blue) and Non-Face (red) FFG timecourses at each visit, with shading showing ± 2 standard errors of the mean. Significant Face vs. Non-Face FFG source strength differences are highlighted (red for M290; blue for M170; gray for M100; using a *p* < 0.05 for 20+ ms family-wise correction).

Figure 3 shows L-FFG and R-FFG source timecourses for the Face and Non-Face conditions as a function of Visit. Within-subject paired *t*-tests comparing Face and Non-Face source strength and conducted separately for each Visit identified Face vs. Non-Face FFG source strength differences, shown *via* the shaded windows (using *p* < 0.05 for 20 + ms family-wise correction). At Visits 1 and 2, larger Face vs. Non-Face FFG responses (i.e., face-sensitive FFG responses) were observed between 250–450 ms post-stimulus. This latency range is consistent with the N290 response latency reported in the infant literature and is thus is defined here as the M290 response (red shading area in Figure 3). With respect to the M290 response, stronger Face vs. Non-Face M290 responses were observed ∼300–450 ms, post-stimulus at Visit 1 in both hemispheres, and ∼250–300 ms post-stimulus at Visit 2 in the right hemisphere. The left-hemisphere magnetic field topography reversed at Visit 2, thus indicating a left-hemisphere change in FFG neural generator activity between ∼18 and 24 months.

At Visits 3 and 4, a more adult-like latency was observed, with the peak face-sensitive FFG response observed at an earlier latency, and thus with these responses defined as the M170. Blue shading indicates when this response was significantly stronger in the Face than Non-Face condition. At Visit 3, in the L-FFG a stronger M170 was observed in the Face than Non-Face condition. Although what appeared to be a R-FFG M170 was observed, as shown in Figure 3, the strength of the R-FFG response did not differ as a function of condition. At Visit 4, a face-sensitive M170 was observed bilaterally, with stronger M170 responses observed in the Face than Non-Face condition between ∼100 and 200 ms post-stimulus.

### The left and right fusiform gyrus peak latency

In Table 2, M290 peak latency values at Visit 1 and Visit 2 are provided for each child. The M290 peak latency score was computed from the Face vs. Non-Face subtraction waveform, first identified as present or absent based on the magnetic field topography (see Figure 2B), and judged present only when the source strength difference waveform had a Z-score > | 1.5| 250–450 ms post-stimulus. When identified as present, the M290 peak latency was scored at the time of the largest Z-score. M170 peak latency was identified at Visits 3 and 4, again first identified as present or absent based on the magnetic field topography, and judged present only when the source strength difference waveform had a Z-score > | 1.5| 100–250 ms post-stimulus. When identified as present, the M170 peak latency was scored at the time of the largest Z-score.

Based on latency, the dominant response at Visits 1 and 2 (< 2 years old) was the M290, with stronger responses (see magnetic field topography in Figure 2B) observed in the Face than Non-Face condition in L-FFG and R-FFG ∼250–450 ms post-stimulus (see Figure 2C, 3; using a *p* < 0.05 for 20+ ms family-wise correction). For some children, M290 appeared to evolve (based on a change in latency) into the M170 at Visit 3 (∼30–36 months), with the L-FFG M170 observed in 64% of children and the R-FFG M170 observed in 85% of children. By Visit 4 (3–4 years), L-FFG and R-FFG face-sensitive responses were more distinct and had more “M170” like latencies (100–250 ms post-stimulus) in > 85% of the children (see Table 2).

**FIGURE 4:**
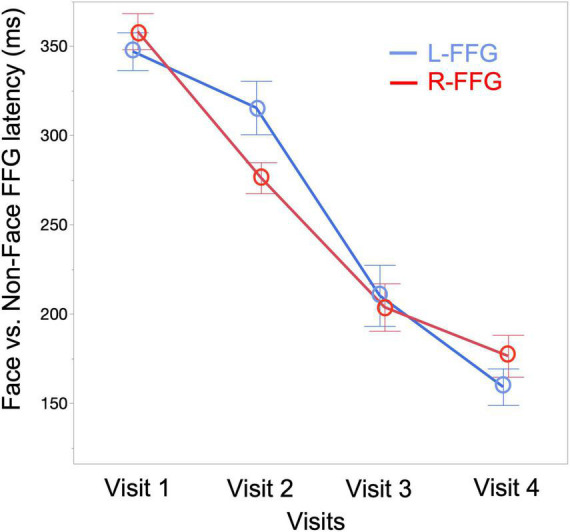
Least-square means plot showing averaged L-FFG (blue) and R-FFG (red) latency at each visit.

To quantify changes to the Face vs. Non-Face FFG peak latency as a function of age, a linear mixed-effects model was run with FFG peak latency as the dependent variable (M290 peak latency for Visit 1 and Visit 2, M170 peak latency for Visit 3 and Visit 4), Visit and Hemisphere and their interaction entered as fixed-effects, and subject as a random-effect variable. Results showed a main effect of Visit [*F*(3, 94) = 100.15, *p* < 0.001], with FFG peak latency decreasing as a function of age. A marginally significant Visit * Hemisphere interaction [*F*(3, 89) = 2.34, *p* = 0.08] was also observed, with simple-effect analyses revealing a later latency in L-FFG than R-FFG at Visit 2 [*t*(19) = –2.27, *p* = 0.02] (see Figure 4 for least-square means plot for averaged L-FFG and R-FFG latency at each visit). No main effect of Hemisphere was observed.

Finally, exploratory chi-square analyses examined the presence vs. absence of a face-sensitive FFG response between hemispheres separately for each visit. In Table 2, the percentage of children showing an M290 or M170 response in each hemisphere is provided under the Visit column. At Visit 1, more Face vs. Non-Face FFG M290 peaks were present in the right than left hemisphere [*X*^2^(1, *N* = *21*) = 5.97, *p* = 0.01]. No hemispheric differences at later Visits were observed.

## Discussion

The present study examined the maturation of face-sensitive FFG responses from 4 months to 4 years. Three main findings were observed. First, whereas the M290 was the dominant face-sensitive response when the children were under 2 years of age, the M170 was dominant when the children were 3–4 years of age. Second, FFG Face vs. Non-Face latencies decreased as a function of age. Third, a right-hemisphere preference to Face vs. Non-Face stimuli was only observed before 12 months of age. The following text discuss these findings with respect to previous research.

### From M290 to M170

Face-sensitive FFG responses were observed ∼300–450 ms post-stimulus at Visit 1 and ∼250–300 ms post-stimulus at Visit 2 (Figure 3). This finding is consistent with EEG findings from Di Lorenzo and colleagues (Di Lorenzo et al., 2020), where a larger N290 response was observed to face vs. house stimuli in the majority of infants at 5 and 10 months of age. By Visit 3, however, stronger face-sensitive L-FFG M170 responses were observed ∼100–250 ms post-stimulus, and by Visit 4 (∼3–4 years) face-sensitive M170 responses were observed in > 85% of the children bilaterally. Present findings showed that between 30 and 36 months of age (Visit 3) a face-sensitive M290 was generally absent, with the M170 clearly observed by 3–4 years old (Visit 4). This pattern may be due to small number of subjects at Time 3, as well as perhaps due to greater between-subject variability in FFG activity between 2.5 and 3 years old.

In cross-sectional studies comparing infants and preschool children, Taylor et al. (1999, 2001) showed that the N290 response observed at 12 months occurs ∼20 ms later than the N170 response observed in 4–5-year-old children (Taylor et al., 1999, 2001). In our previous cross-sectional infant MEG study (Chen et al., 2021), the R-FFG peak latency to face stimuli was observed to decrease from birth to 2 years. In the present longitudinal sample, the FFG Face vs. Non-Face peak latency decreased from 353 ms at Visit 1, to 294 ms at Visit 2, to 206 ms at Visit 3, and to 168 ms at Visit 4 (averaged across subjects and hemispheres at each visit; see Figure 4). Of note, however, is the substantial between-subject variability in FFG maturation, with Table 2 detailing marked individual differences in FFG maturation, and with considerable between-hemisphere as well as between-subject differences in the presence/absence of a face-sensitive M290 or M170 FFG response. In general, however, and consistent with previous research on the maturation of FFG face neural processes (de Haan et al., 2002, 2003; Halit et al., 2003), the present study confirmed the FFG M290 response was the dominant face-sensitive response prior to 2 years of age. Also consistent with previous school-age child studies, by 3 and 4 years old the M170 response was the dominant face-sensitive response (He et al., 2014, 2015). Thus, present findings generally support the hypothesis that the M290 eventually becomes the M170.

### Hemisphere maturation differences

In school-age children and adolescents, a stronger neural response in the right than left hemisphere to face stimuli is often observed (Itier and Taylor, 2004). The developmental origin of a right-hemisphere specialization for face perception is unclear. Whereas some studies have not observed face processing hemisphere differences in infants (de Haan and Nelson, 1999; Tzourio-Mazoyer et al., 2002; Gliga and Dehaene-Lambertz, 2007), other studies have shown a right-hemisphere specialization for face processing during infancy (Le Grand et al., 2003; Tsao et al., 2008; de Heering and Rossion, 2015; Adibpour et al., 2018; Chen et al., 2021). For example, in a cross-sectional study of infants 0–2 years of age (Chen et al., 2021), whereas a Face vs. Non-Face difference in FFG source strength was observed in L-FFG and R-FFG, only R-FFG latency changed as a function of age, and only R-FFG latency was associated with social skills.

Although findings from the present longitudinal cohort provided some evidence of a right-hemispheric specialization for faces, hemisphere findings were not consistent across all ages. In particular, prior to 12 months of age (Visit 1) FFG M290 Face vs. Non-Face differences were more often observed in the right than left hemisphere. This pattern, however, was not observed at other ages, and by 3–4 years old (Visit 4) stronger FFG responses to Face than Non-Face stimuli was observed in both hemispheres. In addition, no hemisphere differences were observed for FFG latency. The present findings are thus generally consistent with previous literature reporting a right-hemispheric advantage in young infants but not in preschool children (Lochy et al., 2020). This may be due to the fact that right-hemisphere face specialization depends on visual discrimination ability. For example, contrary to the face-selective right hemisphere results in infants (de Heering and Rossion, 2015) and adults (Rossion et al., 2015) obtained using the same methodology, Lochy et al. (2019) showed that whereas 5-year-old preschool children show a right-lateralized face response when discriminating upright vs. inverted faces, no lateralized response was observed when viewing faces vs. objects. They concluded that there may be non-linear development of the neural processes underlying face perception, and that hemispheric specialization likely changes across time (e.g., as a result of reading acquisition during preschool years).

Models of hemispheric specialization indicate that whereas the right hemisphere tends to process information in a holistic manner, the left hemisphere tends to process information in a more fine-grained, analytic manner (Banich, 2009). It may be that during infancy, whereas infants rely on the right hemisphere to process faces in a holistic fashion, preschool children rely on both hemispheres to process feature-specific facial information. This is consistent with studies suggesting that different strategies are used at different ages to process featural and configural facial information (Flin, 1985; Baenninger, 1994; Chung and Thomson, 1995), with older children generally exhibiting a left-hemisphere bias when processing the eyes or mouth. It is thus likely that at different ages the FFG processes faces vs. other stimuli differently, and with FFG hemispheric lateralization likely dependent on stimulus conditions (e.g., upright faces vs. inverted faces; faces vs. objects).

### Future directions and limitations

In the present study, the face exam was passive (i.e., no behavioral response) and the Face and Non-Face stimuli were created to be appropriate across the infant to young child age range (Halit et al., 2004) such that any difference in FFG activity between the Face and Non-Face conditions was unlikely to be due to low-level physical properties. A limitation of the present study, however, was the lack of a third stimulus condition that allowed evaluation of FFG responses to holistic and configural face processing (Schwarzer and Roebers, 2002; Schwarzer et al., 2007). Longitudinal birth through preschool and school-age studies are needed to investigate the maturation of FFG holistic vs. configural face processing, as well as FFG activity to specific facial features, such as facial information related to sex and race (Quinn et al., 2002, 2010, 2019). Such studies are difficult, however, as infants, preschoolers, and school-age children have different cognitive, attention, and motor abilities. As such, future studies will need to consider length of task, number of trials per condition, hardware and analyses pipelines, stimuli saliency, whether the stimuli are relevant across a range of ages, and with control conditions that allow for assessment of face, attention, and high- and low-level visual neural processes.

Due to sample size, it was not possible to compare FFG face response maturation in males vs. females, and the present study focused only on FFG activity. Future infant and young child studies examining the maturation of whole-brain face networks in a larger sample of male and females are of interest. Older child and adult functional magnetic resonance imaging (fMRI) and intracranial local field potential studies have identified FFG as the key brain regions active when viewing faces (Allison et al., 1994; Kanwisher et al., 1997; Haxby et al., 1999; Grill-Spector et al., 2004; Kanwisher and Yovel, 2006; Cohen Kadosh et al., 2010; Pourtois et al., 2010). To our knowledge, no fMRI study has examined face encoding during the first few years of life. Although functional near-infrared spectroscopy (fNIRS) has been used to study face processing in infants (Otsuka et al., 2007; Nakato et al., 2009, 2011a,2011b; Ichikawa et al., 2010, 2013; Kobayashi et al., 2011, 2014, 2018; Yamashita et al., 2012; Di Lorenzo et al., 2019), fNIRS lacks the temporal precision and ability to measure activity from deeper cortical regions (Grossmann, 2008; McDonald and Perdue, 2018).

To conclude, study findings showed face-sensitive FFG maturational changes across the first 4 years of life. Whereas a face-sensitive M290 response was observed under 2 years of age (and with a right-hemisphere preference observed before 12 months of age), by 3–4 years old a face-sensitive M170 response was prominent bilaterally. Research evaluating the maturation of face-sensitive FFG activity in infants at risk for neurodevelopmental disorders is of interest, with present findings providing age-specific face-sensitive neural markers as potential prognostic and treatment targets.

## Data availability statement

The original contributions presented in this study are included in the article, further inquiries can be directed to the corresponding author/s.

## Ethics statement

This study was reviewed and approved by the Children’s Hospital of Philadelphia Institutional Review Board. Written informed consent was obtained from the participants’ parent or legal guardian.

## Author contributions

YC, LB, EK, MK, TR, and JE contributed to the conception and design of the study. YC, OA, SL, KM, MS, TC, MK, and EK organized and collected the data. YC and OA analyzed the data and performed the statistical analyses. YC wrote the manuscript. HG and JE wrote sections of the manuscript. All authors contributed to manuscript revision, read, and approved the submitted version.

## Footnotes

1
http://neuroimage.usc.edu/brainstorm
